# Vitamin D3 mediates amelioration of ulcerative colitis via the TRPV1-MAPK signaling pathway

**DOI:** 10.3389/fimmu.2026.1727932

**Published:** 2026-01-30

**Authors:** Keke Zhao, Jian Li, Yersen Asai, Longxiang Zhang, Ziying Hu, Hongliang Gao

**Affiliations:** The First Affiliated Hospital of Xinjiang Medical University, Urumqi, Xinjiang, China

**Keywords:** TRPV1, MAPK, ulcerative colitis, vitamin D3, inflammation

## Abstract

**Introduction:**

Ulcerative colitis (UC), known as a complex inflammatory bowel disease, whose pathogenesis has not been fully clarified. The Transient Receptor Potential Vanilloid 1 (TRPV1), along with its downstream Mitogen-Activated Protein Kinase (MAPK) pathway, are vital in regulating inflammation. However, it remains unclear whether vitamin D3 (VD3) exerts a protective effect by regulating this signaling pathway.

**Methods:**

This study investigated the TRPV1-MAPK pathway in colon tissues from UC patients and in 3% Dextran Sulfate Sodium (DSS)-induced rodents. In vivo experiments were conducted to evaluate the effects of VD3 intervention on disease phenotype, including body weight, Disease Activity Index (DAI), colon length, histological damage, and epithelial cell apoptosis. The study analyzed gene and protein expression levels of TRPV1 and key molecules in the MAPK pathway, and immunofluorescence co-localization was used to assess TRPV1 expression on macrophages. Serum calcium ion (Ca^2+^) levels were measured to explore the modulation of calcium homeostasis.

**Results:**

The TRPV1-MAPK pathway was remarkably upregulated in UC patients and DSS-induced rodents, manifested as upregulated expression levels of TRPV1, phosphorylated p38 (p-p38), and phosphorylated Extracellular Signal-Regulated Kinase (p-ERK). VD3 intervention significantly ameliorated disease phenotype, effectively alleviated weight loss, increased DAI, colon shortening, and tissue damage in mice, and reduced epithelial cell apoptosis. VD3 treatment significantly downregulated both gene transcription and protein expression of TRPV1. Immunofluorescence co-localization confirmed that VD3 reduces TRPV1 expression on macrophages. Alterations in serum Ca^2+^ levels suggested that VD3 may influence TRPV1-mediated calcium influx by modulating calcium homeostasis, thereby suppressing MAPK pathway activation. Consequently, this regulatory cascade led to a significant decrease in pro-inflammatory cytokines, including Interleukin-1Beta (IL-1β), Interleukin-6 (IL-6), and Tumor Necrosis Factor-Alpha (TNF-α), and concurrently restored intestinal barrier function by upregulating the expression of Occludin and Mucin2 (Muc2).

**Discussion:**

This study elucidates a protective role of VD3 in UC, whereby it coordinately regulates immune responses and barrier function via the TRPV1-MAPK signaling pathway, providing a novel theoretical and experimental foundation for VD3-based therapy for UC.

## 1 Introduction

UC is a major subtype of inflammatory bowel disease (IBD), manifesting as a persistent, nonspecific inflammation affecting the colon, with key clinical features such as continuous or recurrent diarrhea, mucopurulent bloody stools, along with abdominal discomfort, and tenesmus as the main symptoms (1, 2). The occurrence of UC is higher in developed countries in Europe and America compared to Asia. Nonetheless, the incidence of UC in Asian regions has considerably increased recently, ranging from 7.6/1000,000 to 14.3/100,000 (3, 4). The exact cause of UC remains unclear and may be closely associated with intestinal immune imbalance, influenced by multiple interacted factors including genetic inheritance, environmental conditions, and intestinal ecological balance (5). Commonly used medications in UC: corticosteroids, 5-aminosalicylic acid, immunosuppressants, biologics, and Janus kinase inhibitors (6). However, a long-term reliance on these medications can result in severe adverse reactions, exorbitant expenses, and a penchant for relapse once they’re discontinued, leading to poor patient compliance and tolerance, posing challenges for treatment (7). Therefore, identifying new drug targets and strategies for UC therapy is crucial.

Recent research indicates that TRPV1 and MAPK play important roles in inflammation regulation. TRPV1 is essential in the nociception system as a non-selective cation channel, which can be activated by various stimuli such as capsaicin, heat, and pro-inflammatory agents, modulating intracellular calcium levels and membrane excitability in many tissues (8). TRPV1 is extensively found throughout the nervous system and in various tissues and organs, present in nearly all mammalian immune cells, for example, the dendritic cells, macrophages, lymphocytes, and neutrophils. What’s more, it is closely related to intestinal inflammation, pain, and visceral hypersensitivity (9). Research indicates that TRPV1 levels are notably higher in UC mice colon tissue, and decreased after sulfasalazine interference (10). Widely present in eukaryotic cells, the MAPK signaling pathway is a significant intracellular signal transduction pathway that includes sub-pathways like Extracellular Signal-Regulated Kinase (ERK), p38, and C-Jun N-terminal Kinase (JNK), which is widely present in eukaryotic cells, participate in the regulation of several biological functions like cell division, maturation, apoptosis, and immune responses (11). In UC, the MAPK signaling pathway is abnormally activated, which subsequently results in phosphorylation and participation in transcribing target genes, protein production, and regulating pro-inflammatory factor secretion. Reducing its activation can effectively reduce inflammatory responses and cell apoptosis induced by UC (12, 13). TRPV1 can be activated by noxious stimuli, which trigger a massive influx of Ca²^+^. As the second messenger, Ca^2+^ initiates a series of intracellular signal transduction events, such as pain, inflammation, and tissue damage (14), potentially forming the TRPV1-MAPK inflammatory axis, which is the key mechanism in UC pathogenesis. Therefore, modulating the TRPV1-MAPK signaling pathway may represent a novel therapeutic strategy for UC.

VD3 denotes a vitamin fat-soluble, serving as a hormonal precursor within the human organism. VD3 is metabolically activated in the liver and kidneys through a two-step process to its active form, 1,25-dihydroxyvitamin D3 (1,25-(OH)_2_D_3_), or the vitamin D hormone, which is the most potent form of VD3 and impacts the human body in numerous ways (15). In recent years, besides its contribution to bone health and the metabolism of calcium and phosphorus, the role of VD3 in regulating the immune system and inhibiting inflammatory responses has received increasing attention. Research suggests that vitamin D deficiency is associated with an increased risk of UC, and VD3 supplementation may have potential therapeutic benefits for UC patients (16). VD3 has an influence on immune cells, including macrophages, dendritic cells, and T cells, affecting the differentiation and activation. The compound 1,25-(OH)_2_D_3_ not only particularly reduces inflammatory reactions, but also strengthens anti-infection mechanisms (17). The specific molecular processes through which VD3 offers protection to the intestines remain unclear. Currently, whether VD3 exerts its intestinal protective effects in UC by modulating the TRPV1-MAPK signaling pathway remains to be elucidated.

The precise mechanism underlying the protective effect of VD3 against UC remains incompletely understood, and existing research has predominantly focused on individual pathways or molecules. To systematically explore the multifaceted role of VD3, this study investigates the potential involvement of the TRPV1-MAPK signaling pathway in mediating the alleviation of UC by VD3. By integrating clinical UC tissue analysis with a DSS-induced murine colitis model, we first confirmed the activated state of the TRPV1-MAPK pathway in UC pathology and subsequently evaluated the therapeutic efficacy of VD3 in ameliorating disease manifestations. Furthermore, through multi-level assessments of molecular expression, functional activity, and upstream-downstream signaling relationships, we demonstrated that VD3 modulates the TRPV1-MAPK pathway, thereby exerting beneficial effects on intestinal inflammation and barrier function. Our findings indicate that the TRPV1-MAPK signaling pathway plays a critical role in VD3-mediated gut protection, providing new experimental evidence for understanding the immunomodulatory function of VD3 and suggesting novel avenues for UC intervention strategies.

## 2 Materials and methods

### 2.1 Human samples

Inflammatory colorectal tissues came from people diagnosed with UC from 2024.1th to 2024.12th (n=5), while the colon tissues of the control group were obtained from patients with colon polyps (n=5). Patients were diagnosed with UC in accordance with the diagnostic criteria set forth in the Chinese clinical practice guideline on the management of ulcerative colitis(2023, Xi’an). UC colon biopsy samples were taken from the ulcer margins in the inflamed regions of the colon, while the control group samples were all colon polyp biopsy samples. This study was approved by the Ethics Committee of the First Affiliated Hospital of Xinjiang Medical University (Ethics approval number: K202501-50). This research protocol was conducted according to the principles outlined in the Declaration of Helsinki. Written consent was obtained from all participants prior to the study.

### 2.2 Animals

SPF grade 8-week-old male C57BL/6 mice, weighing 20 ± 2g, were purchased from the animal center of Newloong Youshu Life Technology (Hangzhou) Co., Ltd. (Animal License No.: SCXK (Zhe) 2023-0012). The rodents were acclimated to their surroundings for a week with a temperature of 22 ± 2°C, relative humidity 40-70%, and a standard light-dark cycle, with no restrictions to hydration and sustenance. Mice were sorted into 3 distinct cohorts (6 members each): Control group, DSS group, and DSS + VD3 group, ensuring balanced weight, age, and other aspects across groups. This study protocol was formally reviewed and approved by the Animal Welfare and Ethics Committee (AWEC) of the Newloong Youshu Life Technology (Hangzhou) Co., Ltd. (Ethics approval number: YS-m202502002). The construction of the animal model and the conduct of the experiments strictly adhered to the ethical guidelines for animal research.

### 2.3 Construction of DSS-induced acute colitis model

The construction procedure started after all mice were adaptively managed for 1 week. The Control group was given unfettered access to distilled water for 1 week, while the DSS group and DSS + VD3 group mice were subjected to 3% DSS solution (Cat# YD08001, MP Biomedicals) for 1 week to induce acute UC model (18). Successful model construction was indicated by significant loss in weight, diarrhea, and bloody stools. During this period, the DSS + VD3 group received intraperitoneal injection of 100 µg/KG VD_3_ solution (Cat# 375320, MCE) on days 0, 2, 4, and 6 (16), while the Control group and DSS group were given a single intraperitoneal injection of an equal dose of solvent on the same days.

### 2.4 Model evaluation

The basic condition of mice was observed and transcribed daily during modeling, including weight loss, bloody/purulent stools, diarrhea, dull fur, arched back, reduced activity, lethargy, loss of appetite, foul-smelling stool, perianal redness and swelling, and increased anal temperature. Significant loss in weight, diarrhea, and bloody stools indicated that the UC model has been correctly constructed.

### 2.5 Disease activity index score

Body weight was measured daily, and mouse behavior, stool consistency, degree of diarrhea, and mortality were observed. DAI was calculated as follows: (a) Stool consistency: 0, well-formed pellets; 2, loose stools; 4, diarrhea; (b) Blood in stool: 0, normal; 2, positive for occult blood; 4, blood stool; (c) Weight loss: 0 (none), 1 (1~1.5%), 2 (5~10%), 3 (10~20%), 4 (> 20%). For each rating, DAI score = a+ b+ c (19).

### 2.6 Fecal occult blood detection by ortho-toluidine method

Fresh feces were collected from each cage on day 7 of modeling. Fecal samples were collected promptly and tested immediately to avoid reduced sensitivity due to prolonged storage and to avoid contact with water, following the instructions of the occult blood test kit (Cat# ml095013, Enzyme-linked Biotechnology). The results analysis is as follows: (a) Negative, no color change within 2 minutes; (b) +, light green gradually turns into green after 10 seconds; (c) 2+, initial light green after the addition of reagent, gradually transforms into blue-brown; (d) 3+, immediate blue-brown after the addition of reagent, gradually transforms into dark brown; (e) 4+, immediate blue-black brown after adding reagent.

### 2.7 Determination of spleen index

Body weight was measured before execution. The spleen was excised after execution, residual blood was blotted with filter paper, and the spleen weight (mg) was recorded. Calculation: Spleen Index = (the weight of spleen/the weight of body) × 10.

### 2.8 HE staining

After 7 days of modeling, execute the rodents, colons were meticulously removed, then photographed and measured at the same time. A 1-centimeter segment of colon tissue will be collected, preserved in 10% neutral formalin solution, embedded in paraffin, sliced routinely, and these sections were subsequently stained using hematoxylin (Cat# CTS-1097, Fuzhou Maixin Biotechnology Development Co., Ltd.) and eosin (Cat# ZLI-9613, Beijing Zhongshan Jinqiao Biotechnology Co., Ltd.).

### 2.9 PAS staining

Tissue samples from colons were preserved in 10% neutral formalin, then we embedded them with paraffin, sectioned routinely, these sections were subsequently stained using periodic acid solution and Schiff’s reagent (Cat# G1008, Servicebio), and then counterstained with hematoxylin to visualize the cell nucleus, and finally dehydrated with anhydrous ethanol and sealed.

### 2.10 TUNEL staining

Tissue samples from colons were preserved in 10% neutral formalin, then we embedded them with paraffin, sectioned routinely, and then we stained them with a TUNEL kit. Samples were analyzed under a fluorescence microscope, with the glass slides protected from light. DAPI (Cat# AR1176, Boster Biological Technology) could staine both apoptotic and non-apoptotic nuclei blue, while the apoptotic cell nucleus is localized by green fluorescence with the incorporation of CF488-dUTP (Cat# G1504, Servicebio).

### 2.11 Immunohistochemistry

Colon collections were preserved in a 10% neutral formalin solution. Subsequently, they underwent embedding, sectioning, baking, deparaffinized, hydration, antigen repair and quenching of endogenous peroxidase, these prepared sections were then exposed to primary antibodies against TRPV1 (ab305299, Abcam), p-p38 (28796-1-AP, Proteintech), p-ERK (28733-1-AP, Proteintech), and Occludin (DF7504, Affinity) in a chilly 4°C environment for a full night. Once the unbound primary antibodies were washed away, the slices were treated with secondary antibodies linked to peroxidase. Staining intensity and extent in tissue sections were observed and scored with an optical-microscope.

### 2.12 Immunofluorescence

Colon samples embedded with Paraffin were made into slices (4 μm thick), deparaffinized, and hydrated throughout an anhydrous ethanol series. The samples were subjected to a 3% BSA blocking solution for a duration of 10 to 30 minutes, incubated with antibodies F4/80 (Cat# 14-4801-82, Thermo Fisher Scientific) and TRPV1 (Cat# DF10320, Affinity) from different species. Sections were counterstained with DAPI to label the cell nucleus after incubation with different fluorescent secondary antibodies, we then examined the stained samples, and took a photograph with a laser confocal microscope. Then measure the fluorescence intensity and the records were subjected to statistical analysis.

### 2.13 Serum Ca^2+^ content

The detection of serum Ca^2+^ was operated with a biochemical kit (Cat# BC0720, Solarbio) following the instructions.

### 2.14 Enzyme-linked immunosorbent assay

The expression conditions of IL-1β, IL-6, and TNF-α in the serum and colon tissue were detected with ELISA kits following the protocol. The kits for IL-1β (Cat# EK201B-96), IL-6 (Cat# EK206-96), and TNF-α (Cat# EK282-96) were all purchased from LiankeBio.

### 2.15 Real-time quantitative PCR

Trizol (Cat# 15596026, Ambion) is used in extracting RNA of the mouse colon, followed by RNA purity evaluation via spectrophotometry. The reverse transcription kit was used in reverse transcription. Then stored cDNA at -80°C. RT-qPCR was performed using SYBR Green PCR premix regent. PCR conditions settings: pre-denaturation (95°C,30 seconds×40 cycles), including denaturation (95°C, 10 seconds), annealing(60°C,20 seconds), extention (70°C, 10 seconds). We then use 2−ΔΔCt method to quantify the expression of relative mRNA. Pimer sequences were as follows:

TRPV1 forward: CCGGCTTTTTGGGAAGGGT, reverse: GAGACAGGTAGGTCCATCCAC; p38 forward: GGCTCGGCACACTGATGAT, reverse: TGGGGTTCCAACGAGTCTTAAA; ERK forward: TCCGCCATGAGAATGTTATAGGC, reverse: GGTGGTGTTGATAAGCAGATTGG; Occludin forward: TTGAAAGTCCACCTCCTTACAGA, reverse: CCGGATAAAAAGAGTACGCTGG; Muc2 forward: AGGGCTCGGAACTCCAGAAA, reverse: CCAGGGAATCGGTAGACATCG; GAPDH forward: AGGTCGGTGTGAACGGATTTG, reverse: TGTAGACCATGTAGTTGAGGTCA.

### 2.16 Western blot

Colon tissue was harvested, and protein concentration was precisely measured using Bicinchoninic Acid (BCA). The exact dosage of the protein sample was then applied to an electrophoresis process and then transferred to a Polyvinylidene Fluoride (PVDF) membrane. Subsequently, these membranes were treated with a thin layer of non-fat milk and then subjected to overnight incubation with primary antibodies against TRPV1 (DF10320, Affinity), p38 (ab170099, Abcam), p-p38 (ab195049, Abcam), ERK (ab184699, Abcam), p-ERK (9101S, CST), Occludin (DF7504, Affinity), and Muc2 (DF8390, Affinity) at a cool 4°C temperature. Once that stage was complete, the membranes were given a wash and followed by another round of incubation, this time with secondary antibodies conjugated with Horseradish Peroxidase (HRP) at room temperature for 80 minutes. Finally, the expression level of protein was observed by an electrochemiluminescence (ECL) system and a gel imaging apparatus.

### 2.17 Statistical analysis

SPSS 26.0 was employed in statistical analysis. Normally distributed metrological data was described as mean ± standard deviation ( $\bar{\text{x}}$ ± s). The independent samples t-test was applied for analyzing the difference between the two groups. The one-way ANOVA was applied for analyzing the difference between multiple groups, with significant variables further compared with the LSD test(homogeneity of variance) or Dunnett’s-T3 test (heterogeneity of variance). Non-normally distributed metrological data were described as M(P25 ~ P75), the Mann-Whitney U test is used for comparison between two groups, and the Kruskal-Wallis H test is used for comparison between multiple groups. A *P* value < 0.05 is deemed statistically significant. All figures were produced with GraphPad Prism 10.0.

## 3 Results

### 3.1 Abnormal activation of the TRPV1-MAPK signaling pathway in the colon tissue of UC patients

**Figure 1:**
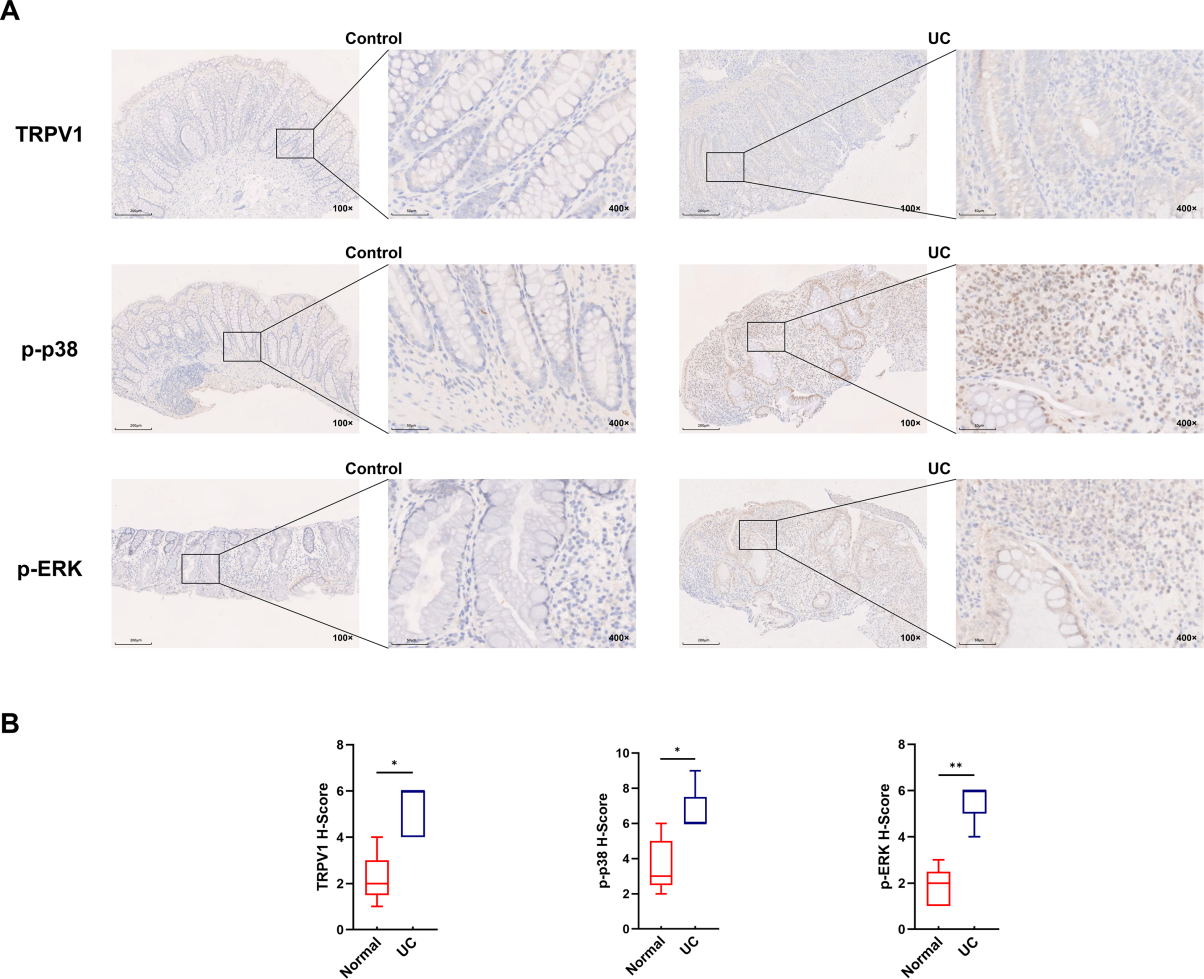
TRPV1-MAPK signaling pathway-related molecules are highly expressed in UC patients. **(A, B)** Localization and expression of TRPV1, p-p38 and p-ERK in colon tissues in the Control group and UC group revealed by immunohistochemistry. (n = 5, *P < 0.05, **P < 0.01).

To explore potential therapeutic strategies for UC, we analyzed how the TRPV1-MAPK pathway activated in the colon tissue of people with UC. This study analyzed colon mucosal biopsy tissues from 5 UC patients and 5 patients with colon polyps using immunohistochemistry. Our findings revealed that, compared with the control group, TRPV1 expression was markedly elevated in UC samples, with positive signals primarily distributed in the mucosal epithelial layer, and more importantly, p-p38 and p-ERK also showed high levels of expression in UC tissues, indicating the MAPK cascade was remarkably activated (**Figure 1A**). Further immunohistochemical analysis demonstrated elevated levels of TRPV1, p-p38, and p-ERK in the UC group compared to the Control group. (**Figure 1B**), These results indicate that TRPV1-MAPK signaling pathway-related molecules are highly expressed in UC patients, suggesting their potential important role in UC inflammation.

### 3.2 VD3 significantly improves the general condition and colon injury in DSS-induced UC mice

#### 3.2.1 VD3 significantly improves the general condition of DSS induced UC mice

**Figure 2:**
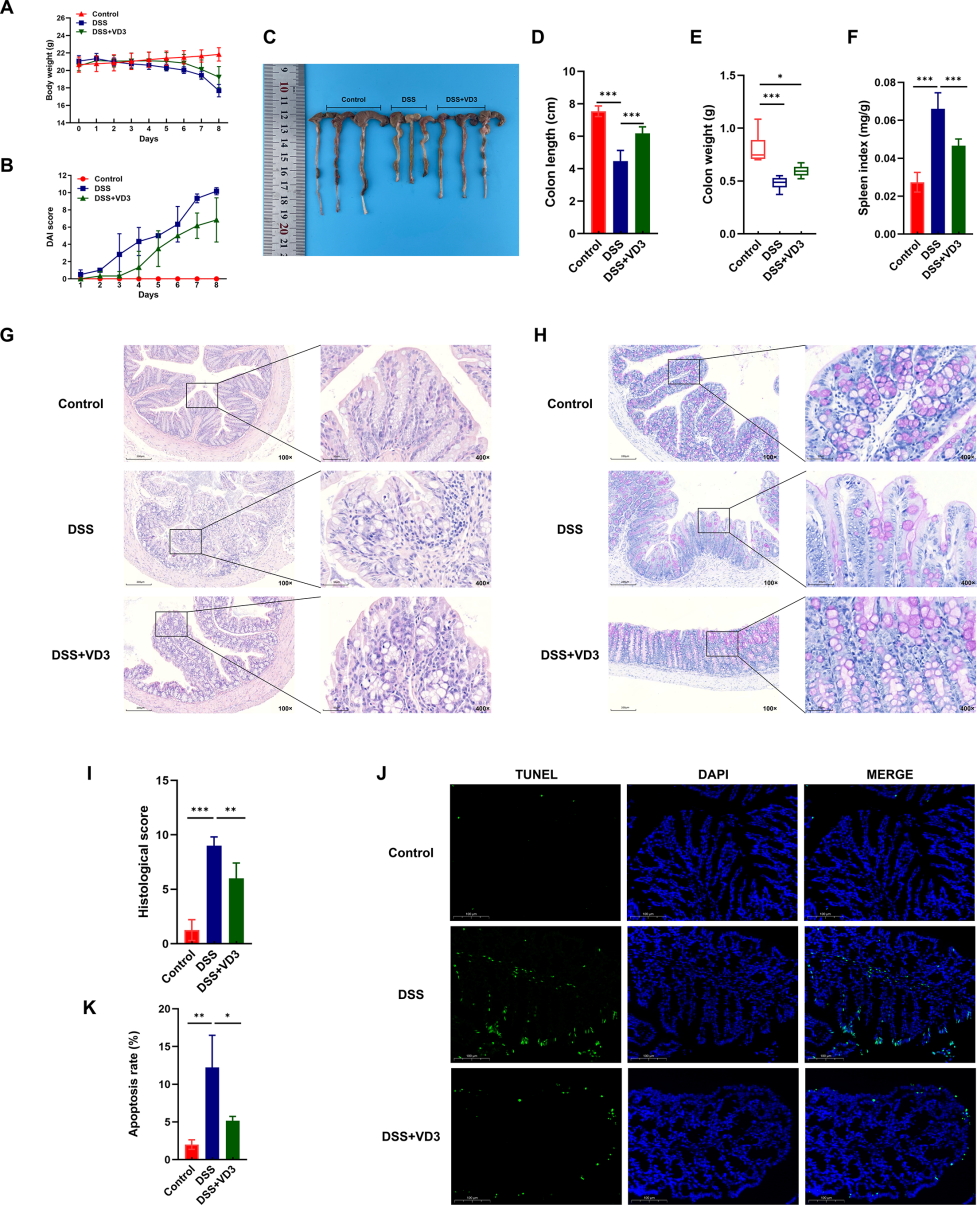
VD3 significantly alleviates clinical manifestation and improves colon injury in UC mice. **(A)** Daily body weight fluctuations across experimental cohorts in a 7-day acute colitis model induced by 3% DSS. **(B)** DAI scores of rodents during experiment. **(C, D)** Variations in colonic length observed among the different groups. **(E)** Comparison of colon weight among groups. **(F)** The spleen index for mice in each group. **(G, I)** HE staining performance and pathological scoring of colon tissues from rodents. **(H)** PAS staining performance of goblet cells in colon tissues from mice in each group. **(J, K)** TUNEL staining performance and analysis of apoptosis rate in colon tissues. (n ≥ 4, *P < 0.05, **P < 0.01, ***P < 0.001.).

This research employed a 3% DSS-induced acute UC rodent model to demonstrate the influences of VD3 on UC progression. During modeling, daily observation revealed that from day 3 onwards, mice within the DSS cohort gradually showed lethargy, decreased activity, decreased food and water intake, bloody stools, and dull fur, while the VD3 intervention group showed improvement in daily status. Furthermore, after DSS induction, mice in the model group showed weight loss, increased DAI scores, and elevated fecal occult blood scores, indicating successful modeling. The VD3+DSS group exhibited a reduction in weight loss, DAI scores, and fecal occult blood scores (**Figures 2A, B**, and **Table 1**). Mice were executed after 7 days of modeling. The DSS group exhibited reductions in both colon length and weight. In contrast, the VD3+DSS group showed a notable recovery in colon length; although the reduction in colon weight was less pronounced, it did not differ significantly from that in the DSS group. In parallel, the Spleen Index rose in the DSS group, whereas VD3 therapy notably curtailed the Spleen Index in the VD3+DSS group (**Figures 2C-F**). These findings suggest that VD3 effectively alleviates the clinical symptoms of UC and reduces colonic inflammation and systemic immune responses.

**Table 1:** Statistical analysis table of fecal occult blood data.

Groups	M (P25 - P75)	Wilcoxon rank-sum test	
		Z	P
DSS	4.00 (4.00, 4.00)	-2.739	0.006
DSS + VD3	3.00 (2.50, 3.25)		

#### 3.2.2 VD3 repairs colonic damage in UC mice

Furthermore, this study conducted histological observations on colon tissue, including HE staining, PAS staining, and TUNEL staining. HE staining revealed disordered colonic structure, crypt damage, inflammatory cell infiltration from the mucosa to the muscularis, and ulcerative necrotic foci in some tissues of the DSS group. Conversely, the VD3+DSS group exhibited relatively intact colonic structure, reduced crypt loss, decreased abnormal hyperplasia, and alleviated inflammatory cell infiltration within both mucosa and submucosa layers. Meanwhile, histopathological evaluation revealed that HE scores were markedly higher in the DSS group, whereas they were notably lower in the VD3+DSS group (**Figures 2G, I**). The staining with PAS showed a reduction in the number of goblet cells, uneven cell morphology and size, and disordered arrangement in the DSS group, while the VD3+DSS group showed significant histological improvement, with increased numbers of goblet cells and more orderly arrangement in the colon tissue (**Figure 2H**). TUNEL staining showed a higher count of positively stained cells in the DSS group, positive cells were not only present at the crypt base but also diffusely distributed in the intestinal glands and mucosal epithelium, with visible positive cell fragments. After VD3 intervention, the count of these positive cells dropped within the colon tissue, positive signals were mainly limited to the crypt base, with improved continuity of the mucosal epithelium. A numerical analysis of the TUNEL staining disclosed that the apoptosis rate was notably elevated in the DSS group than that in the Control group, whereas the rate in the VD3+DSS group was notably diminished compared to the DSS group (**Figures 2J, K**). The above results confirm that VD3 intervention effectively reverses the colonic pathological changes instigated by DSS in UC mice.

### 3.3 VD3 treatment is associated with suppression of the TRPV1-MAPK signaling pathway

#### 3.3.1 Reduced TRPV1 expression and function following VD3 treatment

**Figure 3:**
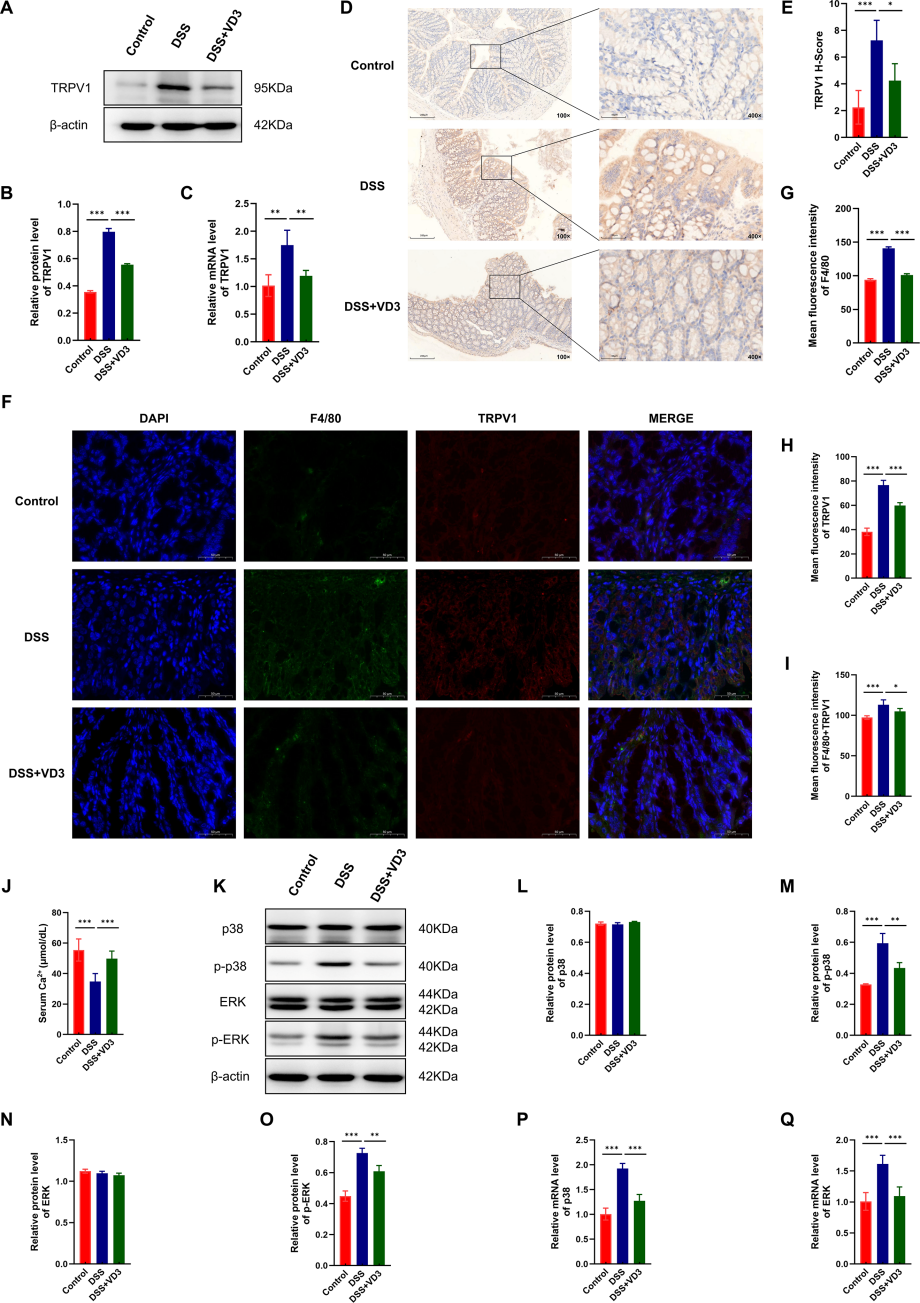
VD3 inhibits the TRPV1-MAPK signaling pathway. **(A)** Protein expression levels of TRPV1 in colon tissues of the VD3-treated UC mouse model detected by Western Blot. **(B, C)** Protein and mRNA expression levels of TRPV1 detected using Western Blot and qRT-PCR. **(D, E)** Immunohistochemical analysis reveals localization and expression patterns of TRPV1 within the colonic tissues of ulcerative colitis mice. **(F-I)** Immunofluorescence expression and quantitative analysis of F4/80, TRPV1, and co-localization of F4/80 with TRPV1 in colon tissues of UC mice. **(J)** Serum calcium ion concentration in each group. **(K)** Protein expression levels of p38, p-p38, ERK, p-ERK in colon tissues of the VD3-treated UC mouse model detected by Western Blot. **(L-O)** p38, phosphorylated p38, ERK, and phosphorylated ERK protein expressions observed via Western Blot. **(P, Q)** mRNA expression levels of p38 and ERK detected by qRT-PCR. (n ≥ 3, *P < 0.05, **P < 0.01, ***P < 0.001.).

To verify whether VD3 targets TRPV1, we first detected TRPV1 expression in colon tissue. The Western Blot analysis demonstrated that, compared to the Control group, the colon tissue from the DSS group sported a notably higher TRPV1 protein expression. After VD3 intervention, TRPV1 protein expression was significantly inhibited (**Figures 3A, B**). qRT-PCR results similarly showed an increase in TRPV1 mRNA transcription in the DSS group, which was notably diminished in the VD3+DSS group, indicating that VD3 negatively regulates TRPV1 at the transcription level (**Figure 3C**).

Additionally, to clarify the localization and cell types expressing TRPV1 in colon tissue, we performed immunohistochemistry and immunofluorescence staining. IHC staining showed that TRPV1 protein predominantly resided in the colon’s mucosal epithelium and submucosa. TRPV1 positive signals were notably enhanced in the DSS group, along with increased numbers of positive cells and expanded expression areas. After VD3 intervention, TRPV1 positive expression in the colon tissue was notably inhibited, with lower staining intensity and positive cell area, as opposed to the DSS group. IHC scoring analysis revealed a significantly higher TRPV1 expression score in the DSS group compared to the control group, while the score in the VD3+DSS group was notably reduced, indicating that VD3 effectively downregulates DSS induced overexpression of TRPV1 protein in the colon (**Figures 3D, E**). Immunofluorescence revealed that F4/80 signals were significantly enhanced and diffusely distributed in the colon tissue of the DSS group, indicating substantial macrophage infiltration and activation due to inflammation. After VD3 intervention, F4/80 signal intensity was significantly reduced. Similarly, TRPV1 signals were significantly enhanced in the DSS group, with expression distribution consistent with IHC results, primarily located in the mucosal layer, and its expression decreased after VD3 intervention. Through immunofluorescence co-localization analysis of F4/80 and TRPV1, we observed a high degree of spatial overlap between F4/80-positive and TRPV1-positive areas in the merged images from the DSS group, indicating that infiltrating macrophages highly co-expressed the TRPV1 channel. After VD3 treatment, the signals in these co-localized areas decreased, and the TRPV1 signal on residual macrophages also diminished. Quantitative analysis of fluorescence intensity showed that compared to the Control group, the average fluorescence intensities of F4/80, TRPV1, and their co-localized signals were remarkably upregulated in the DSS group, and VD3 treatment remarkably reduced these values (**Figures 3F-I**). These results reveal that VD3 may inhibit TRPV1 expression on macrophages, thereby modulating their function and ultimately exerting anti-inflammatory effects.

Simultaneously, we detected serum calcium ion concentration to explore the potential link between TRPV1 ion channel function and systemic calcium metabolism. The serum calcium readings in the DSS group were notably lower compared to the Control group, serum calcium concentration significantly rebounded and recovered to near normal levels (**Figure 3J**). This result suggests that the UC pathological status may lead to calcium homeostasis imbalance, and overactivation of the TRPV1 channel with consequent calcium influx might be one potential cause.

#### 3.3.2 Inhibition of MAPK signaling pathway activation by VD3

To explain the downstream mechanism of VD3, this study assessed the activation state of pivotal molecules within the MAPK signaling cascade, specifically p38 and ERK. Western Blot analysis demonstrated a marked elevation in the levels of phosphorylated p38 and p-ERK in the DSS group colon tissue, as opposed to the Control group. VD3 intervention significantly reduced the protein levels of p-p38 and p-ERK. However, the expression levels of total p38 and total ERK protein showed no statistically significant differences among all groups (**Figures 3K-O**). The qRT-PCR test found that the mRNA transcription levels of p38 and ERK were significantly upregulated in the DSS group compared to the Control group, and VD3 treatment downregulated their expression (**Figures 3P, Q**). The results indicate that VD3 inhibits the phosphorylation-mediated activation of the MAPK cascade.

### 3.4 VD3 reduces inflammatory response and restores intestinal barrier integrity

#### 3.4.1 VD3 inhibits the release of systemic and local inflammatory factors

**Figure 4:**
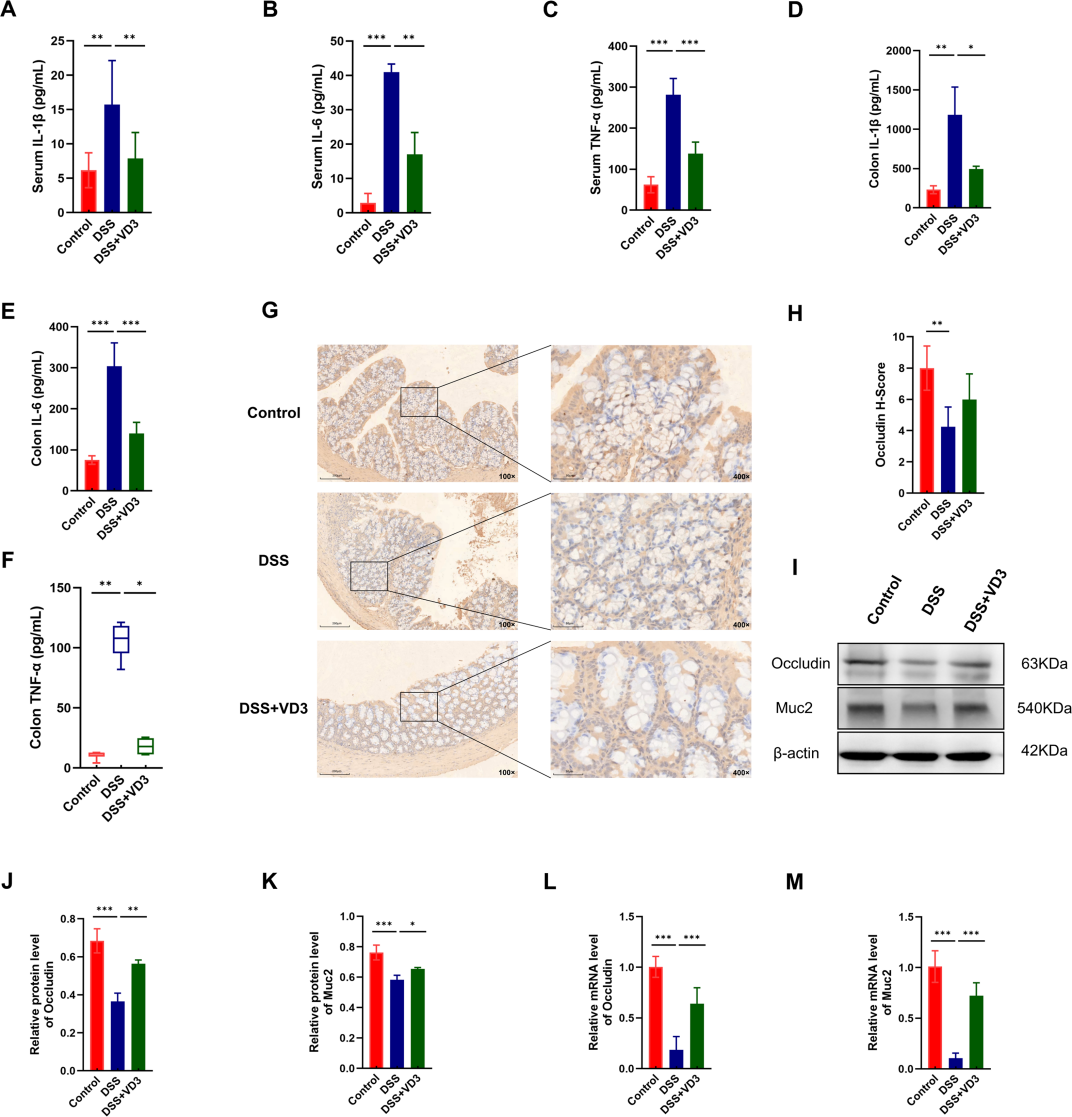
VD3 alleviates inflammation and improves intestinal barrier integrity. **(A-C)** Comparison of plasma concentrations of pro-inflammatory cytokines IL-1β, IL-6, and TNF-α among groups. **(D-F)** Comparison of colon tissue levels of pro-inflammatory cytokines IL-1β, IL-6, and TNF-α among groups. **(G, H)** Localization and expressing levels of Occludin in colon tissues of UC rodents detected by immunohistochemistry. **(I)** Protein expression levels of Occludin and Muc2 in colon tissues of the VD3-treated UC mouse model detected by Western Blot. **(J-M)** Protein and mRNA expression levels of Occludin and Muc2 detected by Western Blot and qRT-PCR. (n ≥ 3, *P < 0.05, **P < 0.01, ***P < 0.001.).

To evaluate the systemic and local anti-inflammatory influences of VD3, we analyzed the concentrations of pivotal pro-inflammatory cytokines IL-1β, IL-6, and TNF-α in mice serum and colon tissue using ELISA. ELISA detection revealed that the levels of key pro-inflammatory cytokines IL-1β, IL-6, and TNF-α were notably elevated in both serum and colon tissue of the DSS group. However, subsequent administration of VD3 led to a remarkable reversal of these cytokine levels, confirming the potent anti-inflammatory effect of VD3 (**Figures 4A-F**).

#### 3.4.2 VD3 repairs the intestinal mucosal barrier

The study hypothesized that reduction of inflammatory response could help alleviate the intestinal mucosal barrier damage due to inflammation. Therefore, we evaluated barrier function related indicators. Immunohistochemistry intuitively revealed the distribution of Occludin protein. In the Control group, Occludin was uniformly and continuously distributed along the cell membrane edges of epithelial cells, forming an intact network structure. In the DSS group, this structure was disrupted, Occludin signals became discontinuous, blurred, and disorderly distributed. After VD3 intervention, the continuous linear distribution of Occludin was reestablished. IHC scoring analysis showed that the Occludin expression score was significantly lower in the DSS group than in the Control group. After VD3 intervention, although the decrease in Occludin expression score was reduced, the discrepancy was not statistically meaningful versus the DSS cohort (**Figures 4G, H**).

For further analysis, we used Western Blot to gauge the expression status of Occludin and Muc2 proteins. Results indicated that both Occludin and Muc2 were notably diminished in the DSS cohort, and VD3 treatment significantly reversed this trend (**Figures 4I-K**). Moreover, our qRT-PCR data revealed that the mRNA transcripts for Occludin and Muc2 were similarly suppressed in the DSS group, and VD3 treatment effectively promoted their gene transcription (**Figures 4L, M**). VD3 can upregulate the expression of Occludin and Muc2, effectively repairing DSS induced intestinal mucosal barrier damage, which is one of its core therapeutic mechanisms in alleviating UC.

To conclude, this research thoroughly investigated the therapeutic potential of VD3 in ulcerative colitis (UC) and shed light on its underlying mechanisms. Further confirmed a pronounced upregulation of the TRPV1-MAPK signaling pathway in UC patients’ colon, suggesting that abnormal activating status of this pathway may play a vital part in the disease’s deterioration. Additionally, the study revealed that VD3 may interfere with TRPV1-mediated calcium signaling, consequently reducing phosphorylation levels in the MAPK signaling pathway. VD3 alleviated intestinal mucosal inflammation and macrophage infiltration, promoted the repair of intestinal barrier function, and ultimately significantly ameliorated the disease severity in the DSS-induced UC rodents by suppressing this pathway. This study provides experimental support for using VD3 as an adjunct or combination therapy for UC, laying an empirical foundation for its clinical application and highlighting the pivotal role of TRPV1 in UC pathology to guide the development of novel treatment strategies.

## 4 Discussion

UC is a chronic disease characterized by recurrent episodes of inflammation, which affects the rectum and colon mucosa, with pathological features including inflammatory damage to the colonic mucosal and submucosal layers, ulcer formation, mucosal surface hemorrhage and desquamation, significantly affecting patients’ quality of life (20). Millions of people around the world suffer from UC, which poses a significant burden on global medical health (21). Therefore, exploring effective treatment strategies for UC and identifying its key regulatory components is of paramount importance. This study confirmed the abnormal activation of the TRPV1-MAPK signaling pathway in colon tissues of UC patients, suggesting its potential role in UC pathogenesis. This finding provides clinical evidence for subsequent mechanistic investigations based on animal models. Therefore, we utilized a DSS-induced UC rodents to investigate the protective functions of VD3 and to uncover the underlying mechanisms involved.

VD3 exerts intestinal protective effects through various pathways, including immune regulation, inflammation suppression, oxidative stress reduction, and fibrosis blockade (22). Epidemiology has confirmed the inversity between serum VD levels and the activation of UC, and vitamin D deficiency allies a higher likelihood of steroid resistance, prolonged hospital stay, and increased operation rates (23, 24). A low 1,25-(OH)2-D3 status can downregulate the expression level of the Colonic Epithelial Vitamin D Receptor (VDR) and proteins Claudin-1, Occludin, and ZO-1, which is related to tight junction, accompanied by increased levels of pro-inflammatory factors TNF-α, IL-17, IL-6, and Hypersensitive C-reactive Protein (hs-CRP), which further increased intestinal barrier permeability and bacterial translocation (25). Furthermore, DSS exerts toxic effects specifically on intestinal epithelial cells, compromising the integrity of the intestinal barrier and igniting inflammation. DSS-induced UC mouse model recapitulates UC symptoms in humans, such as weight loss, diarrhea, bloody stools, mucosal damage, and superficial ulcers, making it widely used for pharmacological evaluation (26, 27). Based on the above research, this study selected the DSS-induced UC mouse model to systematically evaluate the therapeutic effect of VD3 on UC. The results showed that VD3 intervention significantly improved the general condition of DSS mice, reversed colon shortening and splenomegaly, and effectively restored pathological damage in colonic tissue, including reducing mucosal inflammation, restoring epithelial structure, and decreasing epithelial cell apoptosis levels. These outcomes align with previous studies, reinforcing the promise of VD3 as a UC treatment. However, the complete picture of how VD3 alleviates UC remains to be fully elucidated, and continued investigation into its key mechanisms will be essential for building a more robust theoretical foundation for UC clinical management.

To deeply elucidate the molecular mechanism by which VD3 alleviates UC, this study focused on the TRPV1-MAPK signaling pathway, exploring its core function in the anti-inflammatory influences of VD3. Transient Receptor Potential (TRP) channels are a family of non-selective cation channels found abundantly on cell membranes, serving as a pivotal component in the immune inflammatory response and disease progression by regulating inflammatory factors, chemokines, and immune cell activity (28). The sensitivity of these channels are significantly enhanced in inflammatory conditions, making them potential new targets for treating human inflammatory diseases (28). TRPV1 is a well-researched player in the TRP family, which is a non-selective calcium-permeable cation channel widely distributed in various organs, including the nervous system and gastrointestinal tract, and it plays a key role in pain signaling and the control of inflammatory responses (29). Activation of the TRPV1 receptor regulates intracellular calcium concentration, when TRPV1 is activated, it fine-tunes intracellular calcium levels, triggering the release of neuropeptides like Calcitonin Gene-related Peptide (CGRP) and Substance P (SP) from intestinal sensory nerve endings. These neuropeptides subsequently influence vascular smooth muscle activity, promote the proliferation of inflammatory cells, and facilitate the leakage of plasma proteins (30). Numerous studies indicate increased expression of TRPV1 in various inflammatory processes. Overactivation of TRPV1 significantly increases disease susceptibility in DSS-induced colitis mice and promotes the release of pro-inflammatory factors, such as IL-1β and TNF-α (31). Conversely, the application of TRPV1 antagonists or genetically inhibiting this receptor can reduce disease activity and severity and suppress the production of cytokines such as TNF-α, interleukin-2 (IL-2), and interferon-γ(IFN-γ) (32, 33). The protective effects were further validated in TRPV1-deficient mice, which exhibited significantly reduced inflammation and improved disease phenotypes (29, 34). The MAPK cascade activation hinges on ERK phosphorylation and p38 proteins, which activate nuclear transcription factors that govern cell proliferation, stress responses, and inflammatory reactions (35). ERK activation is closely related to pro-inflammatory effects. This pathway can be activated by the upstream protein Ras, which migrates into the nucleus to modulate transcription factors, spark inflammatory responses, and promote cytokine release like TNF-α (36). The p38 MAPK signaling pathway can mediate the production of various inflammatory factors such as TNF-α, IL-1β, IL-6, and IL-8, and participate in processes like apoptosis and neutrophil activation. It also induces intracellular Nitric Oxide (NO) production and boosts Inducible Nitric Oxide Synthase (iNOS) activity, factors intimately connected with UC development (37). Previous research has demonstrated that inhibiting p38 phosphorylation and ERK in the MAPK pathway can markedly cut down pro-inflammatory cytokine expression, such as IL-1β and TNF-α, thereby easing UC symptoms (38). Building on this basis, our study further confirmed that TRPV1 expression is notably upregulated in the colon tissue. Concurrently, the levels of phosphorylated MAPK pathway proteins, p-p38 and p-ERK, are also upregulated. This finding not only aligns with previous reports, but also supports the important role of the TRPV1-MAPK signaling axis in UC pathogenesis from the clinical sample level. The upstream activation of TRPV1 may regulate calcium homeostasis, thereby inducing p38 and ERK phosphorylation, amplifying inflammatory signal transduction, and ultimately causing massive production of pro-inflammatory cytokines and mucosal barrier damage. Therefore, the regulatory role of the TRPV1-MAPK signaling pathway warrants further investigation, as it may offer a potential new direction for UC treatment.

Current evidence suggests that vitamin D may participate in the regulation of this signaling pathway by modulating TRPV1 receptor activity. Previous studies indicate that 25(OH)D and 1,25(OH)_2_D_3_ can bind to the TRPV1 receptor within the capsaicin-binding region. Among them, 25(OH)D has a mild effect on TRPV1 and can greatly reduce capsaicin’s impact on TRPV1, hinting that vitamin D could be a sort of partial stimulator of the TRPV1 receptor, inhibiting its overactivation through negative allosteric modulation. Furthermore, vitamin D can inhibit the overexpression of T cell receptor (TCR)-mediated pro-inflammatory factors TNF-α and IFN-γ by suppressing TRPV1 overactivity (39). Concurrently, vitamin D influences anti-inflammatory actions through the regulation of the MAPK cascade. Existing evidence reveals that vitamin D effectively reduces the release of pro-inflammatory mediators and boosts anti-inflammatory cytokine production by inhibiting phosphorylation of key proteins within the MAPK signaling cascade (40). Through its negative regulation of the MAPK pathway, vitamin D has demonstrated protective effects in various disease models, such as vasculitis and pulmonary fibrosis, further confirming its potential value in inflammation regulation (41, 42). *In vivo* experiments confirmed that VD3 markedly suppresses the abnormal activation of the TRPV1-MAPK cascade in a DSS-induced UC mouse model. q-PCR and Western blot analyses revealed a significant upregulation of TRPV1, p38, and ERK mRNA in the colonic tissues of DSS-induced rodents, alongside increased expression of TRPV1 protein and enhanced phosphorylation of p38 and ERK. VD3 treatment notably inhibited these elevations in expression and activation, although total protein levels of p38 and ERK remained unchanged across groups. Immunohistochemistry further verified that TRPV1 expression was significantly elevated and widespread within inflamed areas of the colonic mucosa in DSS mice, suggesting its activation and involvement in mucosal inflammation in the UC pathological state. VD3 intervention effectively inhibited TRPV1 protein expression, indicating its regulatory role in TRPV1-mediated intestinal inflammatory responses. Additionally, the observed abnormal high expression of key molecules in the TRPV1-MAPK pathway in the DSS mice was highly consistent with the findings in clinical UC patient colon tissue samples, indicating that the overactivation of this pathway plays a key role in the pathogenesis and development of UC. Our findings corroborate existing literature by demonstrating that VD3 may exert its therapeutic effects through modulating aberrant TRPV1 channel activation. Furthermore, VD3 exhibits specific regulatory effects on the MAPK signaling cascade, where it significantly suppresses the phosphorylation of p38 and ERK without altering their total protein expression levels, thereby potentially inhibiting the TRPV1-initiated inflammatory signal transduction cascade.

The activation mechanism of TRPV1 is closely associated with calcium signaling. Upon activation, the TRPV1 channel mediates extracellular Ca^2+^ influx along its electrochemical gradient, leading to elevated intracellular Ca^2+^ levels. Additionally, TRPV1 activation can further stimulate Phospholipase C (PLC) through G Protein-Coupled Receptor (GPCR) signaling, catalyzing the hydrolysis of Phosphatidylinositol 4,5-bisphosphate (PIP_2_) to generate Inositol 1,4,5-trisphosphate (IP_3_) and Diacylglycerol (DAG). IP_3_ binding to IP_3_ receptors on the endoplasmic reticulum promotes calcium release from intracellular stores, further increasing cytosolic Ca^2+^ concentrations (43). Elevated intracellular Ca^2+^ synergizes with DAG to activate classical Protein Kinase C (PKC) isoforms. Activated PKC can directly phosphorylate Raf (Mitogen-Activated Protein Kinase Kinase Kinase, MAPKKK) or activate Ras through other intermediaries, thereby initiating the Raf-MEK-ERK phosphorylation cascade (44). On the other hand, calcium signaling can also trigger ERK phosphorylation through a Calmodulin (CaM)-dependent pathway, wherein Ca^2+^ binding to CaM forms a Ca^2+^/CaM complex that activates the Ras-specific Guanine Nucleotide Exchange Factor (RasGRF), thereby promoting the accumulation of GTP-bound Ras and subsequently triggering the Ca^2+^/CaM–Ras–Raf–MEK–ERK cascade (45). Furthermore, the Ca^2+^/CaM complex may directly or indirectly lead to p38 MAPK phosphorylation by activating upstream kinases such as MAPKKK (Apoptosis Signal-regulating Kinase 1, ASK1) or MAPKK (Mitogen-activated protein kinase kinase 3/6, MKK3/6) (45). Previous studies have also indicated that PKC activation may participate in regulating p38 MAPK phosphorylation (44). These mechanisms collectively demonstrate that TRPV1 activation-induced calcium signaling can simultaneously activate both ERK and p38 MAPK pathways through PKC-dependent and CaM-dependent routes (46, 47). The MAPK pathway is sensitive to elevated intracellular Ca^2+^ levels and integrates pro-inflammatory signals to mediate inflammatory responses (48). These mechanisms provide a molecular basis for understanding how TRPV1 may regulate the MAPK pathway through calcium signaling. It has been reported that alcohol exacerbates Lipopolysaccharide (LPS)-induced calcium influx via TRPV1, promotes phosphorylation of p38 and ERK, and ultimately upregulates the expression of downstream inflammatory factors including IL-1β, TNF-α, and IL-23, thereby aggravating colitis (49). The research revealed a decline in serum calcium in DSS mice, which was effectively reversed by VD3 treatment. This phenomenon suggests that TRPV1 overactivation may be related to systemic calcium homeostasis imbalance. Overactivation of the TRPV1 channel may lead to abnormal calcium influx and intracellular calcium homeostasis imbalance, thereby depleting the body’s calcium reserves, while inflammation-induced mucosal damage also hinders calcium absorption. VD3 may systematically correct calcium homeostasis imbalance through bidirectional regulation by inhibiting TRPV1 activation and promoting intestinal calcium absorption. Based on previous studies, we further speculate that VD3 may alleviate the pathological process of UC by inhibiting TRPV1-mediated calcium influx, downregulating phosphorylation levels of p38 MAPK and ERK, and consequently attenuating downstream inflammatory cascade responses. This finding links local ion channel activity to systemic metabolic homeostasis and further expands our understanding of VD3’s role in immunomodulatory functions.

After verifying VD3’s regulation of molecular expression in the TRPV1-MAPK pathway, we further assessed its impact on cell type-specific expression with immunofluorescence. Macrophages act as key components of the intestinal mucosal immune defense, whose activation and infiltration are important in exacerbating intestinal inflammation in UC (50). Various inflammatory cytokines secreted by macrophages, such as IL-6 and TNF-α, play an important part in UC pathogenesis. IL-6 serves as a primary mediator in inflammation, involved in inflammation and injury processes. It can enhance intestinal epithelial cell permeability, promote macrophage infiltration into inflammatory sites, and further exacerbate UC development (51). TRPV1 receptor expression has been identified in mouse macrophages, with its antagonists effectively suppressing the release of IL-6, IL-1β, interleukin-18 (IL-18), and cyclooxygenase-2 (COX-2) (52, 53). The research indicated that in DSS-induced UC rodents, there was a marked boost in the positive signal strength for the macrophage marker F4/80, the TRPV1 channel protein, and their shared location, and VD3 intervention reversed this phenomenon. The findings imply that VD3 could alleviate intestinal inflammation by preventing macrophages from invading intestinal tissue and by suppressing the expression of TRPV1 on their surfaces. This, in turn, affects the activation of the downstream MAPK cascade, leading to a reduction in releasing inflammatory mediators. These findings provide a theoretical basis for future exploration of whether VD3 exerts its anti-inflammatory effects by modulating TRPV1 expression in macrophages, while also offering new perspectives for therapeutic strategy research in UC.

Based on VD3’s regulatory effect on the upstream signaling pathway, we further explored its impact on downstream effector factors. The release of various inflammatory factors is one of the key pathological mechanisms of UC (54). The severity of UC is closely related to the imbalance between pro-inflammatory and anti-inflammatory factors, where the overexpression of pro-inflammatory cytokines can further aggravate colonic mucosal inflammation (55). TNF-α, as a key pro-inflammatory factor, can rapidly activate the body’s immune defense mechanisms. More importantly, TNF-α can significantly promote the production and release of other pro-inflammatory cytokines such as IL-6 and IL-1β, forming an amplified inflammatory cascade that further exacerbates inflammation severity and tissue damage (36). This study found that VD3 treatment was shown to markedly decrease the levels of pro-inflammatory markers IL-1β, IL-6, and TNF-α in the serum and colon tissues of mice with colitis, suggesting that VD3 could effectively ease DSS-induced intestinal inflammation by dampening key inflammatory signaling pathways. Previous research has demonstrated that activating the TRPV1 channel triggers the MAPK signaling pathway via Ca2+ influx, which in turn elevates pro-inflammatory mediators such as IL-1β and TNF-α (56). Additionally, suppressing the phosphorylation of p38 and ERK proteins within the MAPK pathway has been proven to notably curb production of IL-1β and TNF-α, thereby mitigating progression of ulcerative colitis (38). The results demonstrated a significant association between VD3 treatment and suppression of TRPV1-MAPK pathway activity, along with reduced pro-inflammatory cytokine levels, suggesting that VD3 may modulate inflammatory factor production by affecting the functional state of this signaling axis.

Although the exact cause of UC remains unclear, damage of the intestinal mucosal barrier is recognized as a core process in its development (57). Serving as the body’s frontline defense, this barrier protects against invading pathogens. The main pathological features of its damage include thinning of the protective mucus layer, disruption of tight junction proteins, and damage to the mucosal lining and crypt architecture (58). On one hand, a reduction in intestinal tight junction proteins like Occludin can cause improved epithelial permeability, allowing luminal antigens to translocate to the mucosa and submucosa, activating immune cells and exacerbating the inflammatory response. On the other hand, the loss of mucin Muc2, specifically secreted by goblet cells, can cause structural damage to the mucus layer, weakening its physical barrier function, making pathogenic microorganisms and inflammatory mediators more likely to directly attack epithelial cells, further aggravating colitis (59–61). Therefore, repairing intestinal barrier function has become an important strategy in UC treatment. This research found that VD3 notably mitigated the decrease in both protein and mRNA levels of Occludin and Muc2 within the colon tissue of colitis-affected mice. Immunohistochemical analysis further revealed that VD3 treatment enhanced Occludin protein expression. These results demonstrated that VD3 ameliorates the pathological characteristics of colitis by restoring goblet cell differentiation and preserving intestinal barrier integrity through repair processes. Earlier research has established that elevated levels of TNF-α and IL-1β disrupt tight junction structures, compromising intestinal barrier function (62). The secretion of these inflammatory cytokines is closely linked to activation of the TRPV1-MAPK signaling pathway. Previous studies have demonstrated that p38 MAPK, as a critical downstream component, compromises intestinal barrier integrity and thereby exacerbates UC progression (63). Furthermore, inhibition of p38 MAPK pathway activation has been found to effectively restore the expression of tight junction proteins, including ZO-1, Occludin, and Claudin-1 (64). Therefore, we speculate that the protective effect of VD3 on the intestinal barrier may be related to its regulation of the TRPV1-MAPK signaling pathway. This regulatory effect may involve reducing inflammatory factor release and mitigating adverse effects on tight junction structures, thereby promoting the expression of barrier-related proteins and facilitating the repair process.

This study systematically elucidates a novel molecular mechanism through which VD3 alleviates UC by modulating the TRPV1-MAPK signaling axis, providing important experimental evidence for understanding the immunomodulatory function of VD3 and offering new insights for the development of targeted therapeutic strategies for UC. However, several limitations should be acknowledged and addressed in future research. At the mechanistic level, although our data demonstrate a significant correlation between VD3 treatment and suppression of the TRPV1-MAPK pathway, the absence of functional gain- and loss-of-experiments using TRPV1-specific antagonists or agonists precludes definitive establishment of a causal relationship whereby VD3 directly acts on TRPV1 to regulate downstream MAPK signaling. Further pharmacological validation is required to substantiate this causal link. Regarding clinical controls, the hyperplastic polyp tissues used in this study, while exhibiting relatively normal morphological features, may possess distinct gene expression profiles and a low-grade inflammatory microenvironment. These inherent differences from truly healthy mucosa mean that such tissues do not constitute an ideal biological baseline control, which may somewhat compromise the robustness of our conclusions. Furthermore, the exclusive use of male animals to avoid potential interference from estrogen fluctuations on inflammatory responses precluded investigation of potential sex-dependent differences in VD3 efficacy. Sex-related factors may influence drug response through multiple pathways, including modulation of VD3 metabolism, calcium homeostasis, and immune cell function. Future studies will integrate pharmacological interventions and gene-editing technologies to verify target specificity, seek more representative clinical control samples to enhance robustness, and systematically include female animal models to comprehensively evaluate the general applicability and potential sex-based variations in VD3 therapeutic effects.

## Data Availability

The original contributions presented in the study are included in the article/Supplementary Material. Further inquiries can be directed to the corresponding author.
