# Supplementary material for: Vitamin D3 mediates amelioration of ulcerative colitis via the TRPV1-MAPK signaling pathway
Source: Front Immunol. 2026 Jan 30;17:1727932. doi: 10.3389/fimmu.2026.1727932 (PMC12901333; doi:10.3389/fimmu.2026.1727932)
Supplement: Supplementary file 1 [file DataSheet1.pdf]

Supplementary Material

Supplementary Material

1 Supplementary Figures and Tables

1.1 Supplementary Figures

TRPV1(95 KDa)

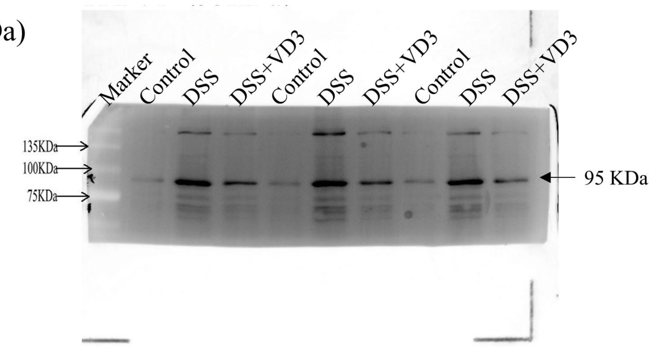

p38(40 KDa)

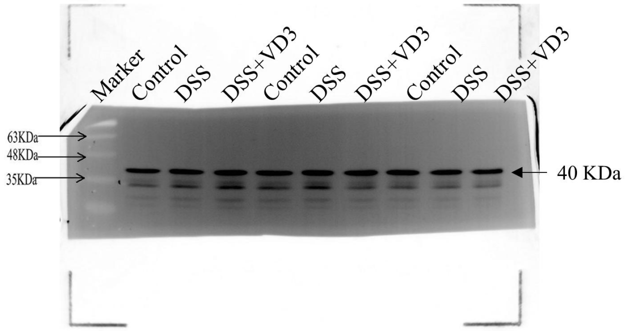

p-p38(40 KDa)

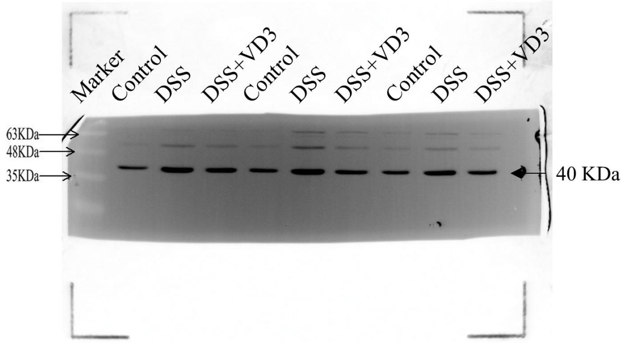

ERK1/2(44/42 KDa)

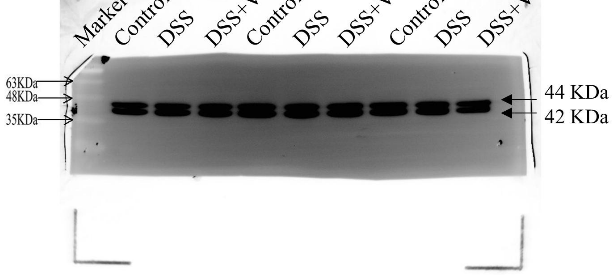

Supplementary Figure 1. Original Western Blot images with white light overlay for TRPV1, p38, phosphorylated p38 (p-p38), and ERK1/2. The images display the detection results of the aforementioned target proteins from top to bottom. Their corresponding representative β-actin loading control images are presented in Supplementary Figure 2 and serve as the loading reference for all target proteins in this figure. Three independent replicate experiments were performed for the detection of each target protein.

p-ERK1/2(44/42 KDa)

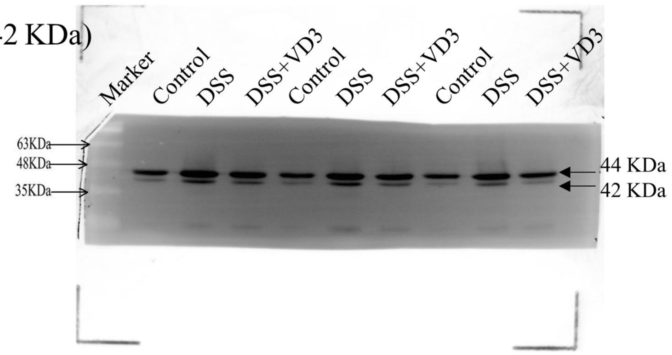

Occludin(63 KDa)

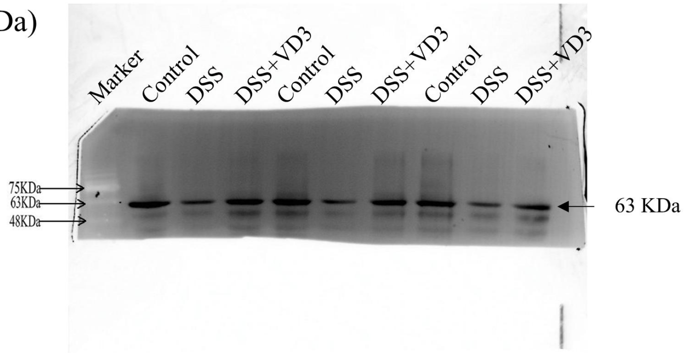

Muc2(540 KDa)

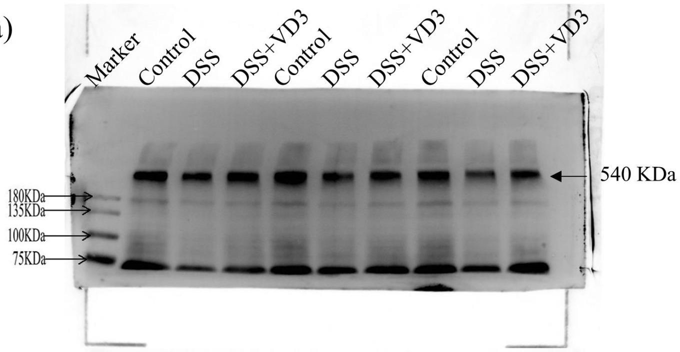

β-actin(42 KDa)

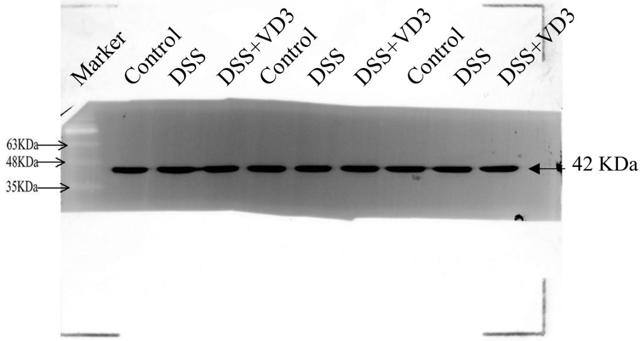

Supplementary Figure 2. Original Western Blot images with white light overlay for p-ERK1/2, Occludin, Muc2, and β-actin. The images display the detection results of the aforementioned target proteins from top to bottom. The β-actin loading control blot shown here is a representative image and serves as the loading reference for all target proteins in this figure and in Supplementary Figure 1. Three independent replicate experiments were performed for the detection of each target protein.
